# Increased frequency of circulating IL-21 producing Th-cells in patients with granulomatosis with polyangiitis (GPA)

**DOI:** 10.1186/ar4247

**Published:** 2013-06-24

**Authors:** Wayel H Abdulahad, Nikola Lepse, Coen A Stegeman, Minke G Huitema, Berber Doornbos-van der Meer, Henko Tadema, Abraham Rutgers, Pieter C Limburg, Cees GM Kallenberg, Peter Heeringa

**Affiliations:** 1 Department of Rheumatology and Clinical Immunology, University of Groningen, University Medical Center Groningen, Hanzeplein 1, 9713 GZ, Groningen, The Netherlands; 2 Department of Pathology and Medical Biology, University of Groningen, University Medical Center Groningen, Hanzeplein 1, 9713 GZ, Groningen, The Netherlands; 3 Department of Nephrology, University of Groningen, University Medical Center Groningen, Hanzeplein 1, 9713 GZ, Groningen, The Netherlands; 4 Department of Laboratory Medicine, University of Groningen, University Medical Center Groningen, Hanzeplein 1, 9713 GZ, Groningen, The Netherlands

## Abstract

**Introduction:**

The present study aimed to explore a possible role for IL-21 producing Th-cells in the immunopathogenesis of granulomatosis with polyangiitis (GPA).

**Methods:**

Peripheral blood from 42 GPA patients in remission and 29 age-matched healthy controls (HCs) were stimulated *in vitro*, and the frequencies of IL-21 producing Th-cells were determined by flow cytometry. Since Th17-cells produce a low level of IL-21, IL-17 was also included in the analysis. Given that IL-21 is a hallmark cytokine for T follicular helper cells (T_FH_), we next evaluated the expression of their key transcription factor BCL-6 by RT-PCR and flow cytometry. To investigate the effect of IL-21 on autoantibody-production, PBMCs from GPA patients were stimulated *in vitro *with BAFF/IL-21 and total IgG and ANCA levels were measured in supernatants. In addition, the expression of IL-21-receptor on B-cells was analyzed.

**Results:**

Percentages of IL-21 producing Th-cells were significantly elevated in GPA-patients compared to HCs, and were restricted to ANCA-positive patients. The expression of BCL-6 was significantly higher in ANCA-positive GPA-patients, as compared with ANCA-negative patients and HCs. IL-21 enhanced the production of IgG and ANCA *in vitro *in stimulated PBMCs from GPA patients. No difference was found in the expression of the IL-21-receptor on B-cells between ANCA-negative patients, ANCA-positive patients, and HCs.

**Conclusion:**

The increased frequency of circulating IL-21 producing Th-cells in ANCA-positive GPA patients and the stimulating capacity of IL-21 on ANCA-production suggest a role for these cells in the immunopathogenesis of GPA. Blockade of IL-21 could constitute a new therapeutic strategy for GPA.

## Introduction

Granulomatosis with polyangiitis (GPA) is an autoimmune vasculitis of small- to medium-sized blood vessels, associated with the presence of circulating anti-neutrophil cytoplasmic autoantibodies (ANCA) that are mainly directed against proteinase 3 [1-3]. Histopathologically, GPA is characterized by granulomatous inflammation and pauci-immune vasculitis, including necrotizing crescentic glomerulonephritis.

Although the production of ANCA is directly attributable to autoreactive B-cells, there is extensive evidence that T-cells play a critical role in GPA as well. The immunoglobulin (Ig)G subclass distribution of ANCA, with a preponderance of the IgG1 and IgG4 subclasses, suggests a T-cell-dependent immune response [4]. Infiltrating T-cells in granulomatous lesions and persistent T-cell activation have been observed in GPA patients [5,6]. In addition, an aberrant T-cell phenotype and impaired regulatory T-cell function are also reported in GPA patients in remission [7-9], suggesting that even during remission, the immune system is dysregulated. Moreover, T-helper (Th) cell polarization with an increase in Th17 cells has been demonstrated [10,11]. Th17 cells and their cytokine IL-17 have been shown to play a critical role in many inflammatory diseases. In addition to IL-17, Th17 cells can produce IL-21, a cytokine that is largely responsible for B-cell class switching and antibody production, and which induces differentiation of B-cells towards plasma cells by synergizing with B-cell activating factor (BAFF)[12,13]. Therefore, it is conceivable that IL-21 may contribute to the production of pathogenic autoantibodies in GPA.

Multiple studies in animal models indicate a pivotal role of IL-21 in the pathogenesis of autoimmune diseases. Studies in arthritis models have shown that blockade of IL-21 activity reduces joint inflammation and destruction [14]. Subsequent investigations demonstrated that blocking of the IL-21 pathway reduces levels of anti-dsDNA autoantibodies and prevents renal disease in mouse models of systemic lupus erythematosus (SLE) [15]. In addition, mice deficient in IL-21-receptor expression were found to be protected to a large extent against the development of inflammatory bowel disease (IBD) and type-I diabetes [16,17]. Interestingly, recent genome-wide association studies have provided convincing evidence that genetic variants in the region on chromosome 4q27 that harbor the IL-21 and IL-2 genes are associated with chronic inflammatory disorders, including SLE, IBD and psoriasis [18-20]. Thus, IL-21 seems to play an important role in autoimmune diseases in general and could constitute a novel target for therapy.

IL-21 is produced mainly by activated CD4^+ ^Th-cells. Recent studies have demonstrated that IL-21, besides its production by Th17 cells, is predominantly secreted by a distinct Th-cell lineage, termed follicular helper T-cells (T_FH_) that express the transcription factor BCL-6 and are considered to be specialized providers of B-cell help [21]. Expansion of circulating T-cells resembling T_FH _cells has been reported in patients with SLE and in patients with rheumatoid arthritis [22-24]. To date, no study has investigated the role of IL-21-producing Th-cells in GPA. Therefore, this study aimed to assess the frequency of IL-21-producing Th-cells, and to evaluate whether T_FH _cells or Th17 cells are the major source of IL-21 in GPA patients. For this purpose, we examined the expression of both IL-21 and IL-17 in circulating CD4^+ ^T-cells of patients with GPA. To improve our understanding of the role of IL-21-producing Th cells in autoantibody production we assessed their frequencies in ANCA-positive and ANCA-negative patients, and studied effects of IL-21 on Ig and ANCA production *in vitro*.

## Methods

### Study population

Forty-two patients with GPA and 29 age- and sex-matched healthy controls (HC) (18 male, 11 female, mean age 56 (SD ± 13) years, range 26 to 72 years, *P *= 0.16) were included in this study. The diagnosis of GPA was established according to the definitions of the Chapel Hill Consensus Conference and patients fulfilled the classification criteria of the American College of Rheumatology (ACR)[25,26]. Only patients without clinical signs and symptoms of active vasculitis and considered to be in complete remission, as indicated by a score of zero on the Birmingham Vasculitis Activity Score (BVAS), were included in the study [27]. Serostatus for ANCA was followed for several months in all patients, and patients with a stable serostatus for ANCA (positive or negative) for at least 3 months were included in this study. Based on these criteria, 23 patients were positive for PR3-ANCA, whereas 19 were ANCA-negative. There was history of generalized disease including renal involvement in 27 patients, and 15 patients had localized disease, which had been confined to the upper and/or lower respiratory tract. None of the patients and controls had infection at the time of sampling. Eleven GPA-patients (eight ANCA-positive, and three ANCA-negative) were treated with maintenance immunosuppressive therapy at the time of blood sampling. Four of them were treated with only azathioprine (25 to 100 mg/day), one patient with mycophenolate mofetil (1500 mg/day), and six patients received prednisolone (5 to 10 mg/day) in combination with azathioprine (125 mg/day). Participants in rituximab trials were excluded from the present study. The main clinical and laboratory data from the patients are summarized in Table 1. All patients and healthy individuals provided informed consent and the study was approved by the local Medical Ethics Committee of the University Medical Centre Groningen, University of Groningen (NL).

**Table 1:** Clinical and laboratory characteristics of patients with granulomatosis with polyangiitis (GPA) at the time of blood sampling

Characteristic	Value
Patients, number	42
Male/female, number	26/16
Age, mean ± SD (range), years	59 ± 14 (28, 81)
Localized/generalized GPA, number	15/27
Positive/negative for PR3-ANCA^†^, number	23/19
Receiving/not receiving treatment^††^, number	11/31
Relapses, median (range), number	0 (0, 5)
Disease duration, median (range), months	112 (20, 334)

^†^Patients were considered to be ANCA-positive when ANCA-titres by IIF were greater than 1:40. ^††^Four patients were treated with azathioprine only, one with mycophenolate mofetil, and six patients received prednisolone in combination with azathioprine. GPA, granulomatosis with polyangiitis; PR3, proteinase 3; ANCA, antineutrophil cytoplasmic antibody.

### Measurement of ANCA titres and specificity

ANCA titers were measured by indirect immunofluorescence (IIF) on ethanol-fixed human granulocytes according to standard procedures as previously described [28]. ANCA titers higher than 1:40 were considered positive. ANCA antigenic specificity was determined using an in-house capture ELISA as described before [29,30]. Briefly, a 96-well plate was coated with goat-anti-mouse Ig for 48 hours. After washing, plates were incubated with mouse monoclonal antibody against human PR3 for 2 hours. After washing, the plate was incubated overnight at 4°C with an extract of human azurophilic granules, which were isolated from neutrophils of healthy donors. Further, serial dilutions of serum (with a starting dilution of 1:100) were incubated for 1 hour. The plate was washed, and the captured antibodies were detected with purified F(ab)_2 _goat-anti-human IgG conjugated to alkaline phosphatase. P-nitrophenyl-phosphate disodium was used as a substrate, and the optical density was measured at 405 nm.

### Antibodies used in flow cytometry

The following conjugated antibodies were used in flow cytometry: allophycocyanin (APC)-conjugated anti-CD3 (clone UCHT1), peridin chlorophyll protein (PerCP)-conjugated anti-CD8 (clone SK1), phycoerythrin (PE)-conjugated anti-IL-21-receptor (clone 17A12), and PerCP-conjugated anti-CD19 (clone 4G7), all purchased from Becton & Dickinson (Amsterdam, The Netherlands); PE-conjugated anti-IL-21 (clone eBio3A3-N2), Alexa Fluor^® ^488 (A488)-conjugated anti-IL-17 (clone eBio64DEC17), and A488-conjugated anti-FoxP3 (clone PCH101), all purchased from eBioscience (San Diego, CA, USA); and PE-conjugated anti-BCL-6 (clone IC5046P) obtained from R&D Systems (Minneapolis, MN, USA).

### Sample preparation and *in vitro *stimulation

Lithium-heparinized venous blood was obtained from patients and healthy donors. Immediately after sampling, 400 μL blood was mixed with 400 μL RPMI1640 (Cambrex Bio Science, Verviers, Belgium), supplemented with 50 μg/ml gentamycin (Gibco, Scotland, UK), and aliquoted in two 5-mL polypropylene tubes (BD Biosciences, Amsterdam, The Netherlands) (400 μL per tube). Diluted blood samples were stimulated 4 hours with 50 ng/mL phorbol myristate acetate (PMA; Sigma-Aldrich, Steinheim, Germany) and 2 nM calcium ionophore (Ca-Io; Sigma-Aldrich). As a negative control, one sample was kept in medium only without stimulation. For inhibiting cytokine release from the cells, 10 μg/ml of brefeldin A (Sigma-Aldrich) was added to each sample.

### Intracellular fluorescence-activated cell sorter (FACS)-staining for cytokines

After stimulation cells were washed in wash buffer (PBS, 5% fetal bovine serum (FBS), 0.1% sodium azide (Merk, Germany)] and stained with PerCP-conjugated anti-CD8 and APC-conjugated anti-CD3 for 15 minutes at room temperature. Cells were fixed with 100 μL Reagent A (Caltag/Invitrogen., Breda, The Netherlands) for 10 minutes. After washing, the pellet was resuspended in 100 μL permeabilization Reagent B (Caltag/Invitrogen) and labeled with A488-conjugated anti-IL-17 and PE-conjugated anti-IL-21 for 20 minutes in the dark. After staining, the cells were washed and immediately analyzed on the FACS-Calibur flow cytometer (Becton & Dickinson). Data were collected for 2 × 10^5 ^cells, and plotted using the Win-List software package (Verity Software House Inc, ME, USA) ME, USA). Because stimulation reduces surface expression of CD4 on T-cells, CD4^+^T-cells were identified indirectly by gating on CD3-positive and CD8-negative lymphocytes. Gated CD4^+ ^T-cells were further displayed as a dot plot for evaluation of intracellular cytokine production. The unstimulated control sample was used as a guide for setting the linear gates to discriminate positive and negative populations.

### Intracellular staining for transcription factors

Peripheral blood mononuclear cells (PBMCs) from GPA patients and HCs were prepared from heparinized venous blood by density-gradient centrifugation on Lymphoprep (Axis-Shield PoC AS, Oslo, Norway). Cells recovered from the gradient interface were washed twice in PBS and stained for BCL-6 and FoxP3 according to the manufacturer's instructions (eBioscience staining set for transcription factors). Briefly, PBMCs were adjusted to 1 × 10^6 ^cells in 100 μL and incubated with appropriate concentration of APC-conjugated anti-CD3 and PerCP-conjugated anti-CD8 for 30 minutes at 4°C in the dark, followed by fixation and permeabilizaion in Fix/Perm buffer (eBioscience) for 45 minutes. Cells were then washed twice with 1 × permeabilization buffer (eBioscience), and stained with PE-conjugated anti-BCL-6 and A488-conjugated anti-FoxP3. After incubation for 30 minutes in the dark, the cell suspension was washed and immediately analyzed on the FACS-Calibur flow cytometer (Becton & Dickinson). Lymphocytes were gated by forward and side scatter patterns, and plotted using the Win-List software package (Verity Software House Inc). Isotype matched control antibodies of irrelevant specificity were obtained from eBioscience and R&D systems.

### Immunofluorescent surface staining for IL-21R on B-cells

Fresh blood samples from GPA patients and HCs were labeled with PE-conjugated anti-IL21R, and PerCP-conjugated anti-CD19 for 15 minutes in the dark. Cells were successively treated with 2 mL diluted FACS lysing solution (BD, Amsterdam, The Netherlands) for 10 minutes and then washed twice in wash buffer and immediately analyzed by flow cytometry.

### RNA isolation and real-time reverse transcription (RT)-PCR

Erythrocytes were lysed and leukocytes were fixed and washed twice in 1% BSA. RNA was isolated from total leukocytes with TRIzol reagent (Invitrogen) according to the manufacturer's instructions. DNAse treatment (Ambion, Huntingdon, Cambridgeshire, UK) was performed and subsequently cDNA was synthesized using M-MLV reverse transcriptase and oligo (dT) 14 to 18 as primer. For measurement of mRNA for BCL-6 and glyceraldehyde-3-phosphate dehydrogenase (GAPDH), 1 μL of cDNA in triplicate was used for amplification by the Taqman RT-PCR system (ABI Prism 7900HT Sequence Detection System, Applied Biosystems, Foster City, CA, USA) with specific Taqman primers/probes (BCL-6 (Hs 00153368_m1) and GAPDH (Hs 99999905_m1), Applied Biosystems). Amplification was performed using standard conditions and calculations of fold induction were performed. We normalized gene expression to GAPDH and expressed values relative to control using the ^ΔΔ^CT (cycle threshold) method.

### Cell stimulation and total IgG production

PBMCs recovered from the gradient interface were washed twice in PBS and adjusted to 10^6 ^cells/mL in RPMI 1640 (Lonza, Basel, Switzerland) supplemented with 10% FCS (Lonza, Switzerland) and 50 μg/mL gentamicin (GIBCO, Invitrogen). Cells were cultured in the presence of 100 ng/mL rhIL-21 (ImmunoTools GmbH, Friesoythe, Germany) and/or 100 ng/mL rhBAFF (PeproTech, NJ, USA) for 12 days at 37°C with 5% CO_2. _After 12 days, culture supernatants were collected and total IgG was measured using an in-house ELISA as described previously [31]. Briefly, Costar 96-well ELISA plates were coated with 2 mg/mL goat anti-human-Ig antibody (Southern Biotech, Birmingham, AL, USA) in carbonate buffer (0.01 M, pH 9.6). Plates were washed with washing buffer (0.025 M Tris-HCl, 0.15 M NaCl, 0.05% Tween-20) and blocked for 1 hour with blocking/incubation-buffer (0.05 M Tris-HCl, 0.3 M NaCl, 0.05% Tween-20, 1% BSA). Cell culture supernatants were diluted in incubation buffer. Purified human IgG with a known concentration was used as a standard sample. The bound IgG was detected with goat-anti-human-IgG antibody conjugated with alkaline phosphatase (Sigma, St Louis, MO, USA). P-nitrophenyl-phosphate disodium was used as substrate and optical density was read at 405 nm using an Emax microplate reader (Molecular Devices, Silicon Valley, CA, USA).

### Measurement of *in vitro *production of PR3-ANCA

*In vitro *PR3-ANCA IgG production in PBMC culture supernatants was measured by Phadia ImmunoCAP^® ^250 analyzer (Thermo Fisher Scientific, MA, USA) using ELiA™ PR3, and the levels of PR3-ANCA IgG production were expressed in response units (RU).

### Statistical analysis

Data are presented as median values unless stated otherwise. The nonparametric Mann-Whitney *U*-test was used to compare data from patients with those of HCs. The Wilcoxon matched pairs test was used for intra-individual comparison. Correlations were assessed using Spearman's rank correlation coefficient. Two-tailed *P*-values lower than 0.05 were considered statistically significant.

## Results

### Increased percentage of circulating IL-21^+^IL-17^- ^cells in ANCA-positive GPA patients compared to ANCA-negative patients and healthy controls

**Figure 1:**
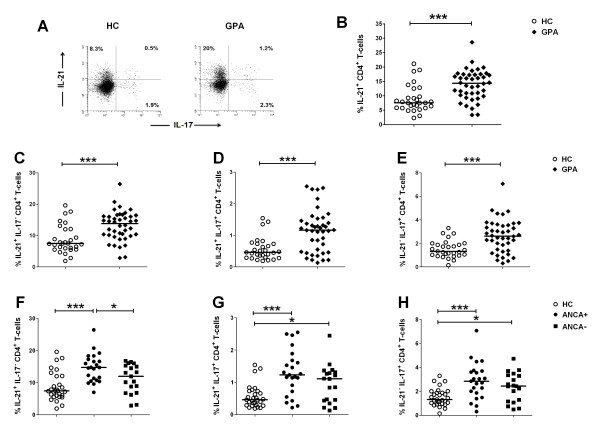
**Multiparameter flow cytometric detection of IL-21 and IL-17 in circulating CD4^+ ^T-cells in patients with granulomatosis with polyangiitis (GPA) and healthy controls (HCs)**. Whole blood from GPA patients and HCs was stimulated with phorbol myristate acetate (PMA)/Ca-ionophore and analyzed for intracellular IL-21 and IL-17 cytokine expression. Representative fluorescence-activated cell sorting (FACS)plots of IL-21 versus IL-17 expression in stimulated CD4^+ ^T-cells from a GPA patient (right plot) and an age- and sex-matched HC (left plot) (**A**). Value in each gate represents the percentages of cytokine producing cells. Percentages of total IL-21-producing Th-cells in peripheral blood of GPA patients (*n *= 42) and HCs (*n *= 29) (**B**). Percentages of circulating IL-21^+^IL-17^-^, IL-21^+^IL-17^+^_, _and IL-21^-^IL-17^+ ^cells within the CD4^+ ^T-cells in all GPA patients and HCs (**C**-**E**), or in antineutrophil cytoplasmic antibody(ANCA)-positive (*n *= 23) and ANCA-negative (*n *= 19) GPA patients (**F**-**H**). Horizontal lines represent the median percentage. *P*-values were calculated using the nonparametric Mann-Whitney *U*-test. *P < 0.05; ***P < 0.0005.

We initially determined the frequency of IL-21-producing CD4 T-cells in the peripheral blood of GPA patients (*n *= 42) and HCs (*n *= 29) after *in vitro *stimulation. The percentage of circulating IL-21^+ ^Th-cells was significantly higher in GPA patients compared with the control group (Figure 1B). Of note, Th17 cells may produce IL-21 in addition to their signature cytokine IL-17. Since Th17 cells are increased in GPA patients [10,11], we next extended our analysis to investigate whether increased IL-21^+ ^Th-cells in GPA patients resulted from an increase in Th17 cells. To this end, IL-17 staining was included in the analysis to determine what percentage of the total IL-21^+ ^Th-cells are Th17 cells. Using this approach, we assessed the frequency of IL-21^+^IL-17^-^, IL-21^+^IL-17^+^, and IL-21^-^IL-17^+ ^cells within the CD4 T-cells in GPA patients and HCs. As shown in Figure (1C, D and 1E), GPA patients in remission had a significantly higher percentages of circulating IL-21^+^IL-17^-^, IL-21^+^IL-17^+^, and IL-21^-^IL-17^+ ^cells compared with the control group. However, the majority of circulating CD4^+ ^T-cells that produced only IL-21 were distinct from Th17 cells, that is, negative for IL-17. To assess the possible role of IL-21^+^IL-17^- ^Th-cells in ANCA production, we compared their percentage between patients who were ANCA-positive (*n *= 23; IIF titer >1:40) or ANCA-negative (*n *= 19) at the time of inclusion. Significant increases in the frequencies of IL-21^+^IL-17^- ^Th-cells were observed in ANCA-positive patients in comparison with HCs and ANCA-negative patients, whereas no significant difference was found between ANCA-negative patients and HCs (Figure 1F). In contrast, the percentages of IL-21^+^IL-17^+ ^and IL-21^-^IL-17^+ ^Th-cells in ANCA-positive GPA patients did not differ from those in ANCA-negative GPA-patients (Figure 1G and 1H). These results suggest that persistence of IL-21^+^IL-17^- ^Th-cells during remission plays a role in the ongoing humoral autoimmune response in ANCA-positive GPA patients.

To rule out the possibility that the increased proportion of IL-21^+^IL-17^- ^Th-cells in GPA patients was the result of current treatment, the ANCA-positive patient group was divided into treated and untreated patients, and the percentages of IL-21^+^IL-17^- ^Th-cells were compared. No significant differences were observed between treated and untreated patients (data not shown). We also compared the percentage of IL-21^+^IL-17^- ^Th-cells between currently untreated ANCA-positive patients with a history of generalized disease and those with localized disease. No difference was found between these patient groups (data not shown).

### Increased frequencies of IL-21^+^IL-17^- ^Th-cells correlate positively with Th17 response

**Figure 2:**
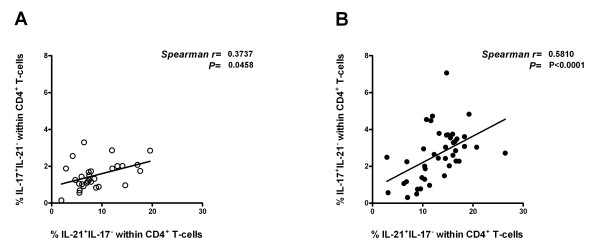
**Correlation between the percentages of IL-21^+^IL-17^- ^cells and IL-21^-^IL-17^+ ^cells within the CD4^+ ^T-cells in peripheral blood of healthy controls (HCs) (A), and patients with granulomatosis with polyangiitis (GPA) (B)**. Spearman rank correlation coefficients (*r*) and *P-*values are given.

It has been reported that IL-21 is a key factor regulating the differentiation of naïve CD4^+ ^T-cells into Th17 cells [32,33]. In order to analyze this relationship, we tested correlation between percentages of IL-21^+^IL-17^- ^Th-cells and percentages of terminally differentiated Th17 cells (IL-21^-^IL-17^+^) in GPA patients (*n *= 42) and HCs (*n *= 29). Interestingly, a significant positive correlation was observed between IL-21^+^IL-17^- ^Th-cells and IL-21^-^IL-17^+ ^Th-cells in both GPA patients and HCs (*r *= 0.58, *P *< 0.0001 and *r *= 0.37, *P *= 0.04, respectively) (Figure 2A and 2B).

### Increased frequencies of BCL-6^+ ^CD4^+ ^T-cells in peripheral blood of ANCA-positive GPA patients

**Figure 3:**
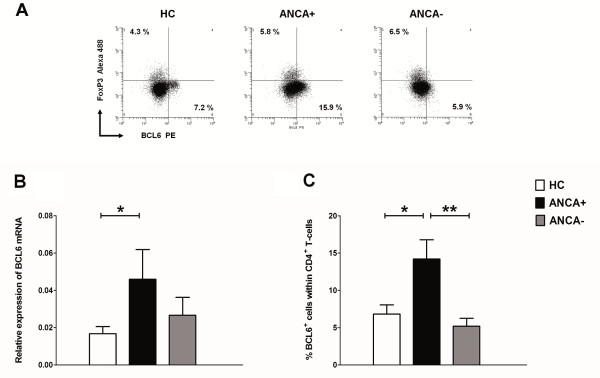
**Expression of transcription factors BCL-6 and FoxP3**. (**A**) Representative FACS plots of BCL-6 versus FoxP3 expression in circulating CD4^+ ^T-cells from an antineutrophil cytoplasmic antibody (ANCA)-negative (right plot) and an ANCA-positive (middle plot) patient with granulomatosis with polyangiitis (GPA), and an age- and sex-matched healthy control (HC) (left plot). Values in each gate represent the percentages of positive cells. (**B**) Relative mRNA expression of BCL-6 in leukocytes from ANCA-positive (*n *= 10) and ANCA-negative (*n *= 6) GPA patients, and age- and sex-matched HCs (*n *= 11) was analyzed by real-time reverse transcription-PCR normalized to the housekeeping gene glyceraldehyde-3-phosphate dehydrogenase (*GAPDH*). (**C**) Percentage of BCL-6^+^FoxP3^- ^cells in circulating CD4^+ ^T-cells was determined in ANCA-positive (*n *= 11) and ANCA-negative (*n *= 10) GPA patients, and age- and sex-matched HCs (*n *= 10). Bars represent the mean values ± SD. *P*-values were calculated using the nonparametric Mann-Whitney *U*-test. *P < 0.05; **P < 0.005.

Since IL-21 is not the only marker for T_FH _cells, we further characterized the identity of circulating IL-21-producing cells by analyzing BCL-6 expression, which is considered a master regulator and specific transcription factor for T_FH _cells [34,35]. To this end, the expression of mRNA BCL-6 was assessed in circulating leukocytes from GPA patients and HCs by real-time RT-PCR. Restricted numbers of patients and controls were included in this analysis due to insufficient cell numbers. Patients with ANCA-positive GPA (*n *= 10) had a significantly higher expression of mRNA BCL-6 than ANCA-negative patients (*n *= 6) and HCs (*n *= 11) (Figure 3B). In addition, intracellular FACS-staining for BCL-6 within circulating CD4+ T cells confirmed the increased BCL-6 expression in ANCA-positive GPA patients (Figure 3A and 3C). We have also analyzed the MFI (mean fluorescence intensity) of BCL-6 expression in CD4^+ ^T-cells from patients and HCs and found that the expression level of BCL-6 per Th-cell in GPA patients was similar to that in HCs (data not shown). Thus, BCL-6 expression is increased in GPA patients due to increased frequencies of circulating BCL-6^+ ^CD4^+ ^T-cells.

Because in recent studies a new population of FoxP3^+ ^regulatory T-cells has been described that shares features with T_FH _cells by expressing the transcription factor BCL-6, we also evaluated whether the increase in BCL-6^+ ^T-cells in GPA patients was a result of an increase in FoxP3^+^BCL-6^+ ^T-cells [36,37]. This analysis showed that the increase in BCL-6 expression in GPA patients was restricted to T_FH _cells and although a low percentage of FoxP3^+^BCL-6^+ ^T-cells was found (< 0.3%), no differences in these cell frequencies were observed between GPA patients and HCs (data not shown).

### Proportions of IL-21-receptor expressing B-cells do not differ between GPA patients and healthy controls

**Figure 4:**
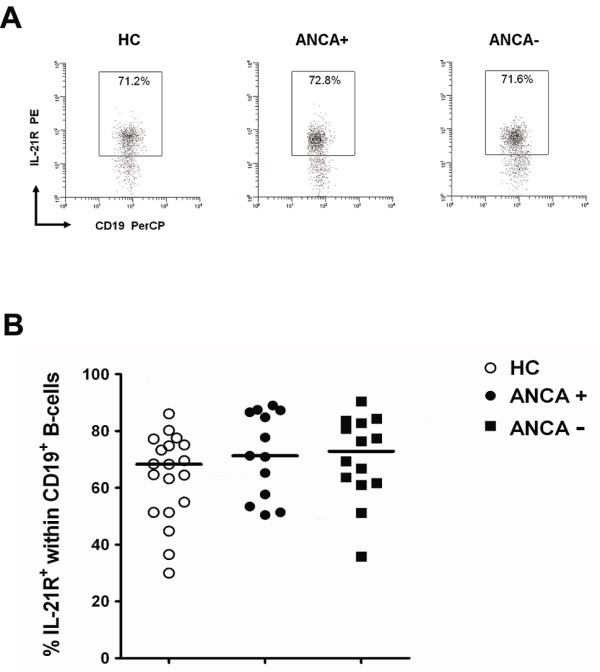
**Comparison of IL-21R-expressing B-cells from patients with granulomatosis with polyangiitis (GPA) and healthy controls (HCs)**. (**A**) Representative fluoresence-activated cell sorting (FACS) plots of IL-21R expression on CD19^+ ^B-cells from an antineutrophil cytoplasmic antibody (ANCA)-negative (right plot) and an ANCA-positive (middle plot) GPA patient, and an age- and sex-matched HC (left plot). Values in each gate represent the percentages of IL-21R^+ ^B-cells. (**B**) The percentage of IL-21R^+ ^B-cells was determined in peripheral blood from ANCA-positive (*n *= 13) and ANCA-negative (*n *= 14) GPA patients, and age- and sex-matched HCs (*n *= 19). Bars represent the mean values ± SD. *P*-values were calculated using the nonparametric Mann-Whitney *U*-test.

Since it is well known that IL-21 acts on B-cells to support their expansion and antibody production [38-40], we conducted further analysis to compare the expression of IL-21R on B cells from GPA patients and HCs. No differences were seen in the percentages of IL-21R^+ ^B-cells either between ANCA-positive (*n *= 13) and ANCA-negative patients (*n *= 14) or between patients and HCs (*n *= 19) (Figure 4).

### IL-21 induces IgG and ANCA production by B-cells from GPA patients

**Figure 5:**
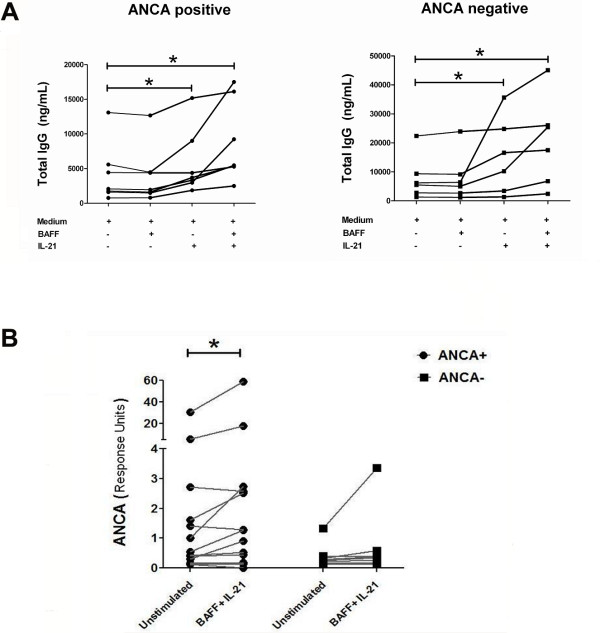
**IL-21 induces *in vitro *immunoglobulin G (IgG) and proteinase 3 (PR3)-antineutrophil cytoplasmic antibody **(**ANCA) production by B-cells from patients with granulomatosis with polyangiitis GPA**. (**A**) PBMCs from ANCA-positive (*n *= 7) and ANCA-negative (*n *= 6) GPA patients were cultured in the presence of rhIL-21 and/or rh B-cell activating factor (BAFF). Culture supernatants were collected after 12 days to measure total IgG by ELISA. (**B**) To assess the effect of IL-21 on *in vitro *ANCA-production, peripheral blood mononuclear ells (PBMCs) from ANCA-positive (*n *= 16) and ANCA-negative (*n *= 12) GPA patients were stimulated in the presence of IL-21/BAFF. After 12 days, PR3-ANCA levels were determined by Phadia ImmunoCAP^® ^250 analyzer. *P*-values were calculated using the Wilcoxon matched pairs test. *P < 0.05.

To explore the interplay between IL-21-producing Th-cells and B-cells in GPA patients, we investigated the effect of IL-21 on IgG antibody-production by B-cells from GPA patients. Restricted numbers of patients and controls were enrolled in this analysis due to insufficient cell numbers. PBMCs from GPA patients were cultured *in vitro *in the presence or absence of exogenous IL-21 for 12 days and total IgG was measured in supernatants by ELISA. Because IL-21 promotes B-cell differentiation by synergizing with BAFF [12,13], we questioned whether the effect of IL-21 on IgG production could be augmented by adding BAFF to the culture. Of note, autologous T-cells in our culture system act as a natural provider of CD40 ligation for B-cells, as this ligation is required for B-cell activation, isotype switching and memory development. As shown in Figure 5, IL-21 significantly enhanced the production of IgG *in vitro *in stimulated PBMCs from both ANCA-positive (*n *= 7) and ANCA-negative (*n *= 6) GPA-patients, whereas stimulation with BAFF alone did not result in increased IgG production. The combination of BAFF and IL-21 tended to increase IgG production more than IL-21 alone. Next, we assessed the effect of IL-21 plus BAFF on *in vitro *production of PR3-ANCA. As shown in Figure 5B, spontaneous PR3-ANCA production was observed in cultured PBMCs from ANCA-positive patients (*n *= 16) in comparison with cells from ANCA-negative patients (*n *= 12). Importantly, IL-21 induces a significant enhancement in PR3-ANCA production in PBMCs isolated from ANCA-positive patients in comparison with ANCA-negative patients. So it is conceivable that autoreactive B-cells were enriched in the peripheral blood of ANCA-positive patients.

## Discussion

In the present study, we demonstrate an increase in the percentage of circulating IL-21-producing Th-cells in GPA patients. We found that elevated frequencies of IL-21-producing Th-cells were restricted to ANCA-positive GPA patients and that these cells were distinct from Th17-cells. We also confirmed that IL-21 can enhance the production of IgG and ANCA *in vitro*.

Over the past few years, Th17-cells have challenged the classical Th1/Th2 paradigm, and have been implicated in a growing number of autoimmune and inflammatory diseases [41]. Recently, a distinct Th-cell subset termed T_FH _and characterized by elevated expression levels of multiple surface proteins and BCL-6 as well as enhanced IL-21 secretion, have been identified as true helper cells for antibody responses. We and others have previously demonstrated that circulating Th17-cells are significantly increased in GPA patients even during quiescent disease [10,11]. However, data are lacking to support a role of IL-21-producing Th-cells in GPA. Since Th17 cells also produce IL-21, we investigated whether Th17 cells in GPA are a source of IL-21. Strikingly, the majority of circulating CD4 T-cells that produced IL-21 were distinct from Th17 cells, indicating that other Th-cell subsets such as T_FH _cells are the source of this cytokine. Importantly, the expansion of T_FH _cells in GPA patients was confirmed by increased BCL-6 expression. To the best of our knowledge, this is the first report demonstrating an increase in the frequency of circulating IL-21-producing Th-cells in GPA, suggesting that T_FH _cell-derived IL-21 may contribute to disease pathogenesis via stimulation of (auto)antibody production.

T_FH _cells are considered to be the major source of IL-21 and seem to be an important subset for adaptive immune responses, although there are conflicting reports on their mode of action *in vivo*. It has been demonstrated that IL-21-producing Th-cells induce Th17 development and proliferation [32,33], which has been shown to promote germinal center (GC) formation in a BXD2 mouse model of autoimmunity [42]. In agreement with these findings, we demonstrate a significant positive relationship between IL-17^+^IL-21^- ^Th-cells and IL-17^-^IL-21^+ ^Th-cells in peripheral blood of GPA patients. It seems likely that increased Th17 cells in GPA-patients are the result of an enhanced T_FH _response, which in turn may participate in granuloma formation and vascular damage. The role of IL-21 in vasculitis was previously suggested by Chen and coworkers [43]. In their study, mice deficient in interferon regulatory factor-4, a protein that inhibits IL-17A production, rapidly developed large-vessel vasculitis and showed increased IL-21 synthesis in addition to increased IL-17A production [43]. Moreover, a role of IL-21 in recruitment of Th17-cells to inflamed tissues has been reported by Caruso and coworkers [44] by showing that IL-21 induces gut epithelial cells to secrete macrophage inflammatory protein-3α (MIP-3α), a chemokine that mediates Th17-cell homing to the skin, joints, and mucosal tissues. Given that endothelial cells are known to produce MIP-3α, it is possible that IL-21 in GPA patients enhances the migration and accumulation of Th17-cells into the vascular wall resulting in inflammation. Besides, IL-21 was shown to enhance granzyme B expression [45] and increase perforin-mediated cytotoxicity by human CD8 T-cells [46] and natural killer cells [47]. It is therefore conceivable that IL-21 can contribute to vessel injury and disease progression in GPA patients. This is an area worth of further investigation.

In contrast to the pro-inflammatory role of T_FH _cells, recent studies have identified a distinctive population of T_FH _cells that displays a regulatory function and suppresses the differentiation of GC B-cells in follicles *in vivo*. This subset was termed follicular regulatory T-cells (T_FR_), which express the regulatory transcription factor *FoxP3 *in addition to their specific lineage transcription factor BCL-6 [36,37]. As circulating *FoxP3*^+ ^T-cells are increased in GPA patients [7], it is conceivable that the observed increase in T_FH _cells in patients is due to an increase in T_FR _cells that co-express *FoxP3 *and BCL-6. We have investigated this possibility but found that the increase in circulating T_FH _cells in GPA patients cannot be explained by increase in T_FR _cells (data not shown).

In our study, increased frequencies of T_FH _cells were observed in patients who were ANCA-positive at the time of inclusion. This suggests the involvement of IL-21 in the process of autoantibody production in GPA. These data are in line with previous reports showing that T_FH _cells act directly on B-cells through the IL-21/IL-21R pathway, and that IL-21 is a potent inducer of class-switch recombination and plasma cell differentiation [39,48,49]. The expression of IL-21R on B-cells from ANCA-positive and ANCA-negative GPA patients was comparable, which suggests that both patient populations have the same ability to respond to IL-21. However, *in vitro *stimulation with IL-21 enhanced the production of ANCA in cell cultures from ANCA-positive patients only, although enhanced total IgG-production was observed in both patient groups. So it is conceivable that autoreactive B-cells were enriched in the peripheral blood of ANCA-positive patients. This might be clinically relevant as well, because ANCA-positive patients are at increased risk for disease relapse [50,51].

In this study, patients were evaluated for the distribution of T_FH _cells during remission. We have previously shown that activated T-cells are present at the time of clinically quiescent disease [9,10]. Furthermore, during active disease effector T-cells appear to migrate towards inflamed tissue [52]. Therefore, in order to study dysbalance of T-cells in GPA patients using peripheral blood samples, we choose to- select patients without or with low dosages of immunosuppressive medication and at the time of clinically quiescent disease.

## Conclusions

In conclusion, the data presented here demonstrate a prominent increase of circulating T_FH _cells in ANCA-positive GPA patients. The key cytokine of these T_FH _cells, that is IL-21, contributes to the production of ANCA autoantibodies *in vitro*. These data support the notion that T_FH _cells are associated with the pathogenic process in GPA patients and may constitute a novel target for therapeutic intervention.

## Abbreviations

ANCA: anti-neutrophil cytoplasmic autoantibodies; A488: Alexa Fluor^® ^488; APC: allophycocyanin; BAFF: B-cell activating factor; BVAS: Birmingham Vasculitis Activity Score; Ca-I: calcium ionophore; ELISA: enzyme-linked immunosorbent assay; FACS: fluorescence-activated cell sorter; FBS: fetal bovine serum; FCS: fetal calf serum; FITC: fluorescein isothiocyanate;T_FH_: follicular helper T-cells; GAPDH: glyceraldehyde-3-phosphate dehydrogenase; GC: germinal center; GPA: granulomatosis with polyangiitis; HC: healthy control; IBD: inflammatory bowel disease; Ig: immunoglobulin; IIF: indirect immunofluorescence; IL-21: interleukin-21; IL-21R: interleukin-21 receptor; MIP-3α: macrophage inflammatory protein-3α; MFI: mean fluorescence intensity; PB: peripheral blood; PBMC: peripheral blood mononuclear cells; PBS: phosphate-buffered saline; PE: Phycoerythrin; PerCP: peridin chlorophyll protein; PMA: phorbol myristate acetate; PR3: proteinase 3; RT-PCR: reverse transcription-polynerase chain reaction; RU: response units; SLE: systemic lupus erythematosus; Th: T-helper; T_FR_: follicular regulatory T-cells.

## Competing interests

The authors declare that they have no competing interests.

## Authors' contributions

All authors contributed to the design, acquisition of data, analysis and interpretation of data. WHA contributed to concept and design, performed the statistical analysis, and had full access to all of the data in the study and takes responsibility for the integrity of the data and the accuracy of the data analysis. WHA, NL, MGH, BDvdM, and HT performed the flow cytometry, *in vitro *experiments, RT-PCR experiments, interpretation of data, and drafting of the manuscript. CAS and AR contributed to concept and design, inclusion of GPA patients, analyses and interpretation of clinical data, and drafting of the manuscript. PCL, PH, and CGMK contributed to concept and design, interpretation of data and revising the manuscript for important intellectual content. All authors read and approved the final manuscript.
